# Multiple Cementoblastoma: A Rare Case Report

**DOI:** 10.1155/2013/828373

**Published:** 2013-08-21

**Authors:** G. Iannaci, R. Luise, G. Iezzi, A. Piattelli, A. Salierno

**Affiliations:** ^1^Department of Pathology, Incurabili Hospital ASLNAPOLI1 Centro, Via Maria Longo, 50-80138 Napoli, Italy; ^2^Department of Medical, Oral and Biotechnological Sciences, University of Chieti-Pescara, Chieti, Italy; ^3^Department of Oral Medicine, School of Dentistry of the Second University of the Study of Naples, Naples, Italy

## Abstract

Benign cementoblastoma is a rare ectomesenchymal odontogenic tumor that originates from the root of the tooth and that is characterized by the formation of cementum-like tissue. A 60-year old man was referred to us complaining of pain in his right jaw. The patient underwent TC dental scan of the mandible, which highlighted the presence of three well-circumscribed, round, unilocular neoformations of radiopaque appearance with a radiotransparent edge, one of which was in close contact with the roots of the lower right second molar. Microscopic examination of the greater sample consisted, in its central portion, of dense mineralized acellular trabeculae of basophilic tissue cement-like, devoid of vessels, adhering to the root of the tooth, while peripherally was observed a zone of vascularized osteoid surrounded, occasionally, by a thin rim of cementoblasts mixed with fibrous tissue and inflammatory elements. This lesion was diagnosed as cementoblastoma. The second lesion appeared radiologically and histologically entirely identical to cementoblastoma, but it did not show the intimate association with the root of involved tooth. After a careful review of the literature, the diagnosis of residual cementoblastoma was made. The clinicopathologic features, treatment, and prognosis of this rare tumor are here discussed for the young dental practitioner.

## 1. Introduction

Benign cementoblastoma is a rare lesion of the oral cavity, currently classified by the World Health Organization (WHO 2005) as an ectomesenchymal odontogenic tumor that originates from the root of the tooth and that is characterized with the formation of cementum-like tissue [1, 2]. It usually arises in the first permanent molars in their mandibular region but can also be associated with multiple teeth, deciduous teeth, or unerupted molars [3, 4]. The caucasians race and male sex are more commonly affected than black race and female (ratio 2.1 : 1), with a very wide age range and a peak incidence between the second and third decade of life. Clinically, the lesion presents as a nodular formation, hard-elastic in consistency producing swelling in the alveolar ridge area. Paresthesias of the lower lip or a pathologic fracture of the jaw have been rarely reported. The radiological findings show a well-defined radiopaque mass surrounded with a thin, radiolucent rim of nonmineralized tissue, in intimate association of the root of the involved tooth. The resorption of the tooth root, the loss of the regular outline with the obliteration of the periodontal ligament are clinical and radiological features that can be frequently found [5, 6]. We describe a rare case of multiple cementoblastoma: a classic cementoblastoma in direct *continuity* with the *root* of the *tooth* (true cementoma) and a residual cementoblastoma localized in the *edentulous area* post-extraction.

## 2. Case Report 

**Figure 1:**
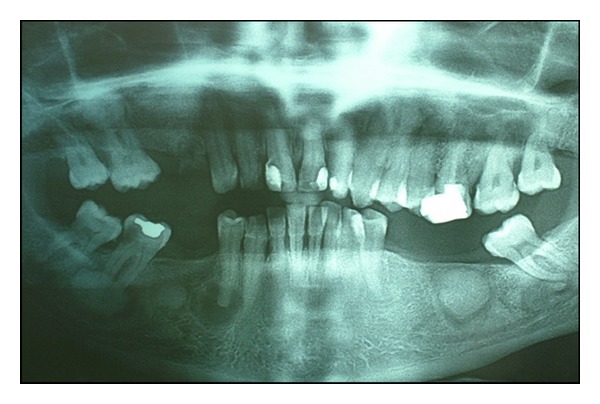
The first orthopantomography revealed the presence of three well-circumscribed, round, unilocular neoformations of radiopaque appearance with a radiotransparent edge, one of which was in close contact with the roots of the lower right second molar.

A 60-years-old man was referred to us complaining of pain in his right jaw. The *examination of the patient's medical history revealed* good general health, absence of systemic diseases, and smoking habit (10 cigarettes∖day). The clinical examination showed discrete oral hygiene, thick and flat periodontal biotype, class II malocclusion with marked loss of vertical size, multiple missing teeth in the site 1.4-1.5-3.5-3.6-3.7-4.5-4.6., presence of swelling, hard elastic consistency, and crepitus on palpation in the region corresponding to the elements 4.4-4.7 and in the edentulous area 4.6, 4.5. The orthopantomography revealed on the upper jaw radicular element in 1.4 region, conservative restoration in 1.1-2.1-2.2-2.6, the presence of radiopaque unilocular lesion adjacent to the root apex of the element 1.6 while the lower arch showed a carious radiolucency at the crown of element 4.7 and other lesions spread to the mandibular body. Particularly, these lesions were thus located: three unilocular round shaped radiopaque lesions with a perilesional radio transparent rib next to the edentulous sites 3.6-4.6 and in contact with the roots of the element 4.7, whose appearance is suggestive for a neoformation (Figure 1); a rounded radiolucent lesion with radiopaque perilesional flange at the root apex of the element 4.4 whose appearance argued for a radicular cyst of endodontic origin. The test thermal pulp vitality with ethyl chloride, performed on elements 4.4 and 4.7, was negative. 

**Figure 2:**
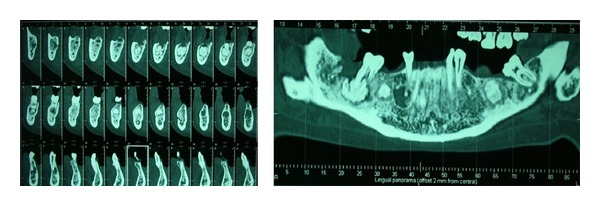
TC dental scan of the mandible.

The initial treatment plan included the implementation of an etiological instrumental therapy (motivation to oral hygiene, periodontal probing, scaling, and radicular smoothing), extraction of root element in endodontic treatment on site 1.4, a conservative endodontic treatment of the element 4.4 in order to resolve endodontically periapical radiolucent lesion and endodontic treatment of 4.7 to cure the acute pulpitis. After two months, following the resolution of acute symptomatology, in order to better analyze the lesions that were evident in the orthopantomography, the patient underwent TC dental scan of the mandible (Figure 2), which highlighted the presence of three well-circumscribed, round, unilocular neoformations of radiopaque appearance with a radiotransparent edge, one of which was in close contact with the roots of the lower right second molar. 

**Figure 3:**
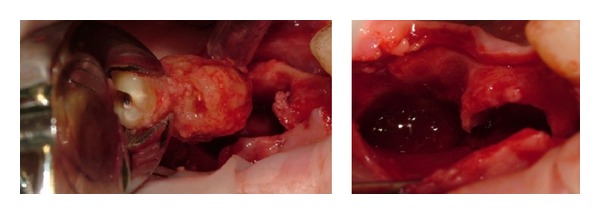
The surgical treatment included the full enucleation of the lesion on site 4.7 with the annexed dental element on the right jaw and the lesion corresponding to the edentulous area 4.6.

The surgical treatment that included the enucleation of the only two lesions on the right jaw, the ones involved in the acute pain, was performed in local and regional *anaesthesia* with a full-thickness flap, osteotomy using rotating tools, *enucleation* of the lesion corresponding to the edentulous area 4.6 and the full enucleation on site 4.7 with the annexed dental element (Figure 3) because the indissociability of the lesion from the root of the dental element has not allowed a more conservative approach “apicoectomy.” Finally after a cleaning of the residual cavity, the flap suture with continuous suture ethicon “3–0” was performed.

**Figure 4:**
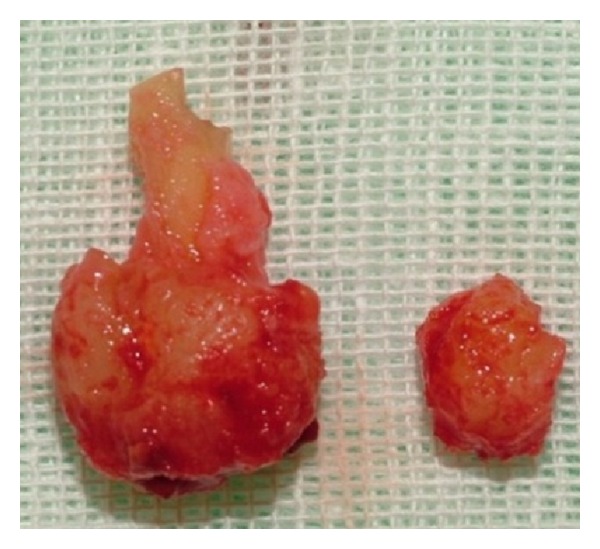
Macroscopically the two samples presented as a nodular, hard-elastic in consistency, the largest of which was adherent to the dental element.

**Figure 5:**
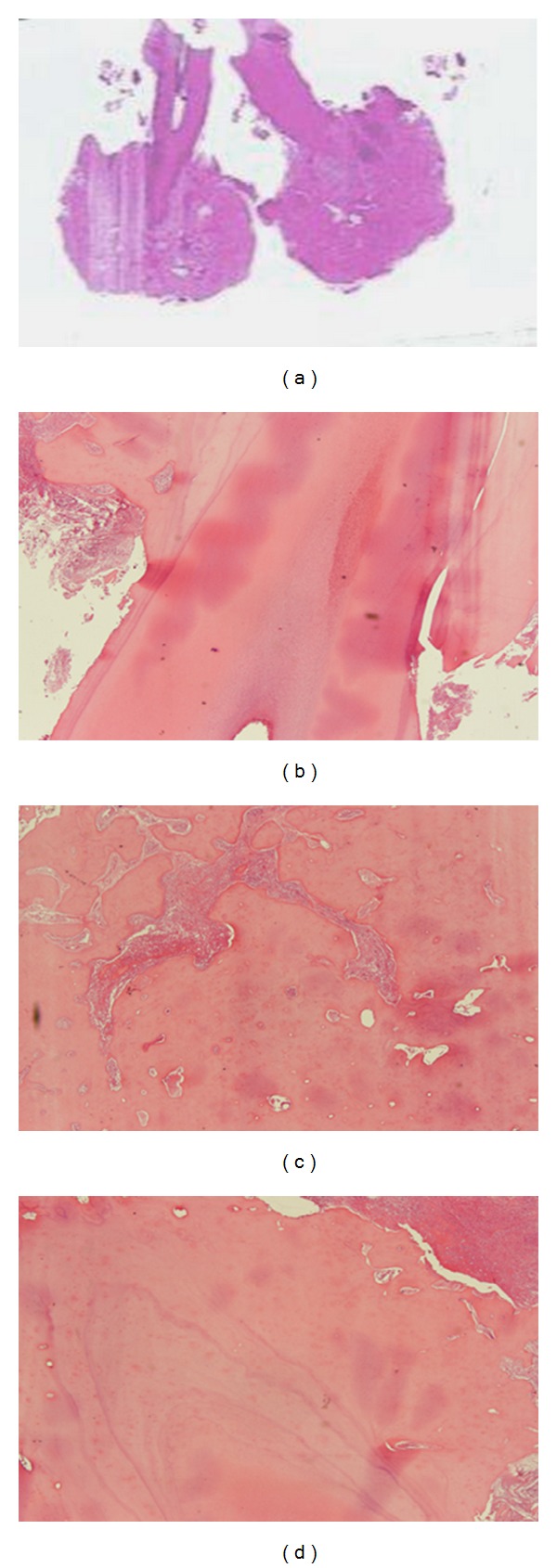
((a) and (b)) H&E (4x∖10x) cementoblastoma closely connected with the tooth root; ((c) and (d)) H&E 20x. Cementoblastoma and residual cementoblastoma revealed the same histological appearance: dense mineralized acellular trabeculae of basophilic tissue cement-like, devoid of vessels, adhering to the root of the tooth.

**Figure 6:**
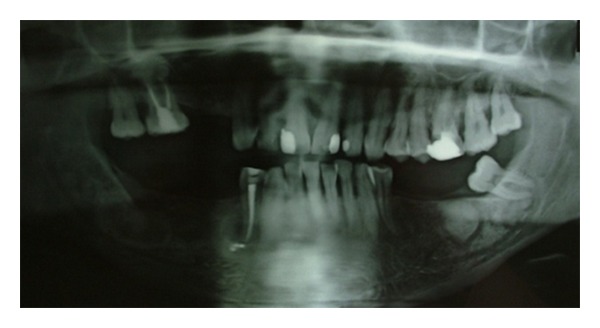
The follow-up after one year of both tumors was negative for disease recurrence.

The specimens were sent to surgical pathology for definitive diagnosis. Both lesions were examined in toto (Figure 4) and the two samples, macroscopically, presented as a nodular, hard-elastic in consistency, color greyish white of 2 × 1 cm and 1 × 1 cm, the largest of which was adherent to the dental element. The tissue samples were fixed in 10% formalin, decalcified with formic acid, and then routinely processed and embedded in paraffin, with cut sections of 3-4 micron. The sections were stained with haematoxylin-eosin. Microscopic examination of the greater sample consisted, in its central portion, of dense mineralized acellular trabeculae of basophilic tissue cement-like, devoid of vessels, adhering to the root of the tooth, while peripherally was observed a zone of vascularized osteoid surrounded, occasionally, by a thin rim of cementoblasts mixed with fibrous tissue and inflammatory elements. The largest lesion, closely connected with the tooth root, was diagnosed as cementoblastoma (Figure 5). The second lesion appeared radiologically and histologically entirely identical to cementoblastoma, but it did not show the intimate association with the root of involved tooth, and, so, it posed the differential diagnosis between osteoblastoma and residual cementoblastoma because both lesions may arise in the edentulous area after extraction. Therefore, after a careful review of the literature and considering the epidemiological and clinical data, the diagnosis of residual cementoblastoma rather than osteoblastoma was made. The clinicopathologic features, treatment, and prognosis are discussed. It is in general agreement with observations emerging from the international scientific literature, which claims that, if the cementoblastoma is properly treated, it does not recur. The follow-up after one year of both tumors was negative for disease recurrence. The features of the lesion remained in site were unchanged (Figure 6). Although this neoplasm is rare, the dental practitioner should be aware of the clinical and radiographic features that will lead to its early diagnosis and treatment.

## 3. Discussion

The location and the histological presentation of benign cementoblastoma is totally identical to osteoblastoma [7, 8]. The osteoblastoma is a rare benign tumor that produces bone, in which the rim of osteoblasts surrounds the trabeculae forming a well-circumscribed lesion, usually greater than 2 cm in diameter. This neoplasm affects young patients; in 90% of cases, they are males under 30 years and the most typical localization includes the mandible: preferentially in the body rather than in the middle portion or in the coronoid process. Radiologically as a well-circumscribed lesion with a mixed pattern, lytic, and sclerotic appears, which reflects the different degree of mineralization of the matrix. It is believed that the two lesions are manifestations of the same process and that the use of a term rather than another is a purely academic exercise [9–11]. Indeed, according to the recent literature, the only difference consists in the fact that osteoblastoma does not melt at the root of the involved tooth as in the case of cementoblastoma that, sometimes, can also involve the periodontal ligament. Other authors, such as Slootweg, classify as osteoblastoma the lesion correlated with root canal but not fused with it. In our case, the second lesion posed further problems of differential diagnosis: in fact it is not possible to value the relationship with the tooth root because the tumour arises in the edentulous area after extraction. Benign cementoblastoma must be, also, differentiated from nonneoplastic processes such as osteoid osteoma that, however, is easily distinguished from a microscopic point of view because it presents a reversed architecture compared to it, presenting dense trabeculae of osteoid in the center rather than peripheral area [12]. After evaluating the site, the patient's age, and rarity in the literature of synchronous association cementoblastoma with osteoblastoma, we opted for the diagnosis of multiple cementoblastoma.
